# Cannabinoid CB_1_ Receptor Agonists Do Not Decrease, but may Increase Acoustic Trauma-Induced Tinnitus in Rats

**DOI:** 10.3389/fneur.2015.00060

**Published:** 2015-03-18

**Authors:** Yiwen Zheng, Peter Reid, Paul F. Smith

**Affiliations:** ^1^Department of Pharmacology and Toxicology, School of Medical Sciences, University of Otago, Dunedin, New Zealand; ^2^Brain Health Research Centre, School of Medical Sciences, University of Otago, Dunedin, New Zealand

**Keywords:** tinnitus, acoustic trauma, cannabinoids, delta-9-tetrahydrocannabinol, cannabidiol, rat

## Abstract

Tinnitus has been suggested to arise from neuronal hyperactivity in auditory areas of the brain, and anti-epileptic drugs are sometimes used to provide relief from tinnitus. Recently, the anti-epileptic properties of the cannabinoid drugs have gained increasing interest; however, the use of cannabinoids as a form of treatment for tinnitus is controversial. In this study, we tested whether a combination of delta-9-tetrahydrocannabinol (delta-9-THC) and cannabidiol (CBD), delivered in a 1:1 ratio, could affect tinnitus perception in a rat model of acoustic trauma-induced tinnitus. Following sham treatment or acoustic trauma, the animals were divided into the following groups: (1) sham (i.e., no acoustic trauma) with vehicle treatment; (2) sham with drug treatment (i.e., delta-9-THC + CBD); (3) acoustic trauma-exposed exhibiting tinnitus, with drug treatment; and (4) acoustic trauma-exposed exhibiting no tinnitus, with drug treatment. The animals received either the vehicle or the cannabinoid drugs every day, 30 min before the tinnitus behavioral testing. Acoustic trauma caused a significant increase in the auditory brainstem response (ABR) thresholds in the exposed animals, indicating hearing loss; however, there was a partial recovery over 6 months. Acoustic trauma did not always result in tinnitus; however, among those that did exhibit tinnitus, some of them had tinnitus at multiple frequencies while others had it only at a single frequency. The cannabinoids significantly increased the number of tinnitus animals in the exposed-tinnitus group, but not in the sham group. The results suggest that cannabinoids may promote the development of tinnitus, especially when there is pre-existing hearing damage.

## Introduction

Tinnitus is the perception and conscious awareness of sound that is not physically present. These phantom sounds can be ringing or buzzing noises or sometimes hissing, grinding, or roaring. Many people experience tinnitus transiently at some time in their life, but for chronic tinnitus sufferers, the condition can be frustrating and debilitating. In severe cases, it can be extremely disturbing, and even lead to suicide (1). Tinnitus affects 25% of the American population at some stage in their life, with 8% of people experiencing persistent or chronic tinnitus (1). While the prevalence of chronic tinnitus normally increases with age, it is alarming that an increasing number of adolescents and young adults are experiencing it due to risky music-listening behaviors, such as prolonged exposure to high-volume music by using portable music players, or going to excessively loud nightclubs or attending pop/rock concerts (2).

Tinnitus can be caused by exposure to loud noise, as well as head and neck injuries; it can also develop as a result of inner ear infection, drug toxicity (e.g., aminoglycoside antibiotics), or as a result of aging (3, 4). Although the mechanisms underlying tinnitus are still not fully understood, the most likely cause of tinnitus is changes in neural activity in the brain, which is supported by both animal and human studies. In animals and humans with tinnitus, neurons in multiple areas of the brain become more active and more neurons fire at the same time in order to compensate for the hearing loss due to damage to the cochlear hair cells (5). Based on the idea that tinnitus is generated by neuronal hyperactivity in the brain, non-benzodiazepine anti-epileptic drugs, such as carbamazepine, are often prescribed [see Ref. (6, 7) for reviews]. However, the preclinical evidence supporting the use of such drugs is limited and contradictory, and the few clinical trials that have been conducted have yielded inconsistent results [see Ref. (4, 6–8) for reviews]. There is also evidence that cannabinoids can suppress epileptiform and seizure activity in animals (9–11). However, there has been no controlled study in humans of the effects of *Cannabis* or cannabinoids on tinnitus itself.

One problem is that *Cannabis* contains over 400 different chemicals, with 66 cannabinoid chemicals unique to the genus. Studies in neuropharmacology have tended to focus on the key psychoactive ingredient, delta-9-tetrahydrocannabinol (delta-9-THC); however, there are many other cannabinoids in *Cannabis* such as cannabinol and cannabidiol (CBD), and it is not always obvious which cannabinoid is exerting the observed effects. In addition to synthetic cannabinoid receptor agonists, such as dronabinol and nabilone, which are used clinically for the treatment of nausea, vomiting, and wasting, natural *Cannabis* extracts such as a 1:1 ratio of delta-9-THC and CBD (Sativex™), are used for the treatment of spasticity and chronic pain in multiple sclerosis (12).

There are two classes of cannabinoid receptors, the CB_1_ and CB_2_ receptors. The general consensus is that CB_1_ receptors are expressed mainly in the CNS, while the CB_2_ receptors are localized mainly to the immune system, peripheral nervous system, testes, and retina [see Ref. (13) for a review]. The presynaptic localization of many CB_1_ receptors and their inhibition of calcium influx at presynaptic terminals may be the basis for any anticonvulsant effects, depending on the neurotransmitter being released. Both Zheng et al. (14) and Tzounopoulos et al. (15) quantified CB_1_ receptor expression in the cochlear nuclei. Tzounopoulos et al. (15) observed CB_1_ receptors in the dorsal cochlear nucleus (DCN) at the parallel fiber/cartwheel cell, parallel fiber/fusiform cell synapses, and on the dendritic spines of cartwheel cells, using electron microscopy. Furthermore, Zhao et al. (16) demonstrated that both fusiform and cartwheel cells expressed diacylglycerol (DAG) α and β, the two enzymes necessary for the production of the endocannabinoid, 2-arachidonyl glycerol (2-AG). Therefore, there is substantial evidence for an endocannabinoid system within the DCN, which may be important for the development of tinnitus.

Only two studies to date have investigated the relationship between CB_1_ receptors in the CN and tinnitus. Zheng et al. (14) studied the expression of CB_1_ receptors in the DCN and ventral cochlear nucleus (VCN) of rats in which tinnitus had been induced using salicylate injections. They found a significant decrease in the number of neurons expressing CB_1_ receptors in the VCN compared to control animals. In the only animal study of the effects of cannabinoids on tinnitus itself, Zheng et al. (17) investigated the effects of two CB_1_ receptor agonists, WIN55,212-2 and CP-55940, on tinnitus induced by salicylate injections in rats. Neither WIN55,212-2 nor CP55,940 significantly reduced the conditioned behavior associated with tinnitus perception. However, 3 mg/kg WIN55,212-2 and 0.3 mg/kg CP-55940 did significantly increase this behavior in normal control animals, suggesting that these cannabinoids might induce tinnitus-related behavior.

Given the lack of evidence relating to the effects of *Cannabis* on tinnitus in humans and the recent data supporting the existence of an endocannabinoid system in the cochlear nucleus, the aim of this study was to further investigate the effects of cannabinoid drugs on acoustic trauma-induced tinnitus, using a 1:1 ratio of delta-9-THC and CBD, which is equivalent to Sativex™ used in the treatment of spasticity and chronic pain in multiple sclerosis (12).

## Materials and Methods

### Animals

Fifty male Wistar rats (300–350 g at the beginning of the experiments) were used in this study. The animals were housed in groups of 2–3 per cage under a 12:12 h light:dark cycle at 22°C and were water deprived throughout the tinnitus behavioral testing. All procedures were approved by the University of Otago Committee on Ethics in the Care and Use of Laboratory Animals.

### Drugs

Delta-9-THC and CBD were purchased from THC Pharm GmbH (Frankfurt, Germany). The drugs were dissolved in Tween 80 and Ethanol (1:1) to make a 50 mg/ml stock solution of the mixture of delta-9-THC and CBD. A working solution containing 1 mg/ml of delta-9-THC and 1 mg/ml of CBD was made freshly every day by diluting the stock solution with saline. This 1:1 ratio of delta-9-THC and CBD was designed to approximate the cannabinoid drug, Sativex™, which is used in the treatment of spasticity and chronic pain in multiple sclerosis in humans (12). Multiple doses of this mixture were not tested simply due to the expense of the drugs.

### Experimental design

The animals were randomly divided into sham (*n* = 20) and exposed (*n* = 30) groups and exposed to either the acoustic trauma or sham procedure. One month later, the animals were tested for the behavioral signs of tinnitus using a conditioned lick suppression paradigm. Following the confirmation of tinnitus, the acoustic trauma-exposed animals were further divided into exposed-tinnitus and exposed-no tinnitus groups. The effects of cannabinoids on tinnitus were investigated by administering either vehicle or delta-9-THC (1.5 mg/kg, s.c.) and CBD (1.5 mg/kg, s.c.) every day, 30 min before tinnitus testing, throughout the tinnitus testing period for a total of 27 days. These doses were the maximum doses that could be used without causing sedation in rats, during a pilot study. The animals were then given a 2-week washout period for the drugs to be eliminated before being tested again for the behavioral signs of tinnitus.

### Acoustic trauma to induce tinnitus

The animals were exposed to unilateral acoustic trauma using the methods described in our previous publications (18–22). Briefly, the animals were anesthetized with a fentanyl (0.2 mg/kg, s.c.) and medetomidine hydrochloride (0.5 mg/kg, s.c.) mixture and placed inside a sound attenuation chamber. A 16 kHz pure tone with an intensity of 115 dB, generated by a NI 4461 Dynamic Signal Acquisition and Generation system (National Instruments New Zealand Ltd.), was delivered to one of the ears for 1 h through a closed field magnetic speaker with a tapered tip (Tucker-Davis Technologies). The unexposed ear was blocked with cone-shaped foam and taped against the foam surface inside the sound attenuation chamber. The sham animals received the same anesthetics and were kept under anesthesia for the same duration as the acoustic trauma animals, but without acoustic trauma exposure.

### Hearing levels

Hearing levels were measured using auditory brainstem response (ABR) thresholds in both the ears of exposed and sham animals before the acoustic trauma, in both the ears of the exposed animals immediately after the acoustic trauma, in the ipsilateral ear of all exposed animals and in both ears of selected sham animals at the conclusion of the study. Briefly, the animals were anesthetized as previously described and acoustic stimuli were presented directly to the entrance of the ear canal using the same set-up as for the acoustic trauma. Stainless steel needle electrodes were placed s.c. at the vertex and over the bullae with a reference electrode at the occiput. ABR thresholds were tested for tone bursts presented at a rate of 50/s. Tone bursts (2 ms rise/decay, 1 ms plateau) were presented in a decreasing intensity series, beginning with levels that elicited distinct evoked potentials. Hearing thresholds were indicated by the lowest intensity that produced visually distinct potentials, progressing in 20-, 10-, and 5-dB steps for 8, 16, 20, and 32 kHz stimuli (18–22).

### Behavioral assessment of tinnitus

The presence of tinnitus was assessed using a conditioned lick suppression paradigm as described in our previous publications (18–22). Briefly, the animals were water deprived and allowed to drink inside an operant conditioning test chamber (ENV-007, Med Associates Inc.) by licking through a sipper tube. The number of licks was sensed by an infrared photobeam and recorded on a computer. The animal’s free-feeding weight was taken as a baseline and the body weight was monitored every day before the behavioral testing. If a rat made less than 1000 licks during any given session, extra water was provided for 30 min in its home cage after the testing session and if there was a weight loss of 10% of their baseline body weight, extra water was provided outside the testing period. This water deprivation schedule typically kept their body weight at 90–95% of their baseline body weight and motivated the rats to produce reliable licks (1500–3500 licks per session) during the tinnitus testing sessions.

The conditioned lick suppression paradigm consisted of 15 min of testing every day and the animals went through three phases: the acclimation phase, the Pavlovian conditioned suppression training phase, and the frequency discrimination phase. During the acclimation phase, a broadband noise (BBN, 60 dB SPL) was presented throughout the 15 min session except at 10 random intervals, at which point 15 s acoustic stimuli presentations were inserted. Two of the 10 presentations were always speaker off periods (i.e., silence) and the remaining 8 were either BBN, 20 kHz tones or 32 kHz tones at 4 different intensity levels (30, 40, 50, and 70 dB SPL for BBN; 70, 80, 90, and 100 dB SPL for 20 and 32 kHz) in a random order with each stimulus repeated twice within each session. The type of stimulus was varied randomly between sessions, but remained constant within a session, and the stimulus presentations did not occur within 1 min of one another, or within 1 min of the beginning or the end of the session. The animals had three sessions of acclimation for each type of stimulus.

Following acclimation, each animal received conditioned suppression training in which a 3 s foot shock (0.35 mA) was presented at the end of each speaker off (silence) period. Over a few sessions, the animals learned the association between the speaker off and the foot shock and reacted to the speaker off by stopping licking. The number of licks in the 15 s period preceding the stimulus presentation and the number of licks during the 15 s of the stimulus presentation were recorded. The lick suppression was measured by comparing the number of licks in these two periods, i.e., the suppression ratio (SR):

$$\mathrm{SR}=\frac{B}{A+B}$$

where *A* is the number of licks in the preceding period and *B* is the number of licks in the stimulus presentation period. If a rat did not make any licks during the 15 s period preceding the stimulus presentation, the corresponding SR for this particular period was excluded.

Once the lick suppression was established (SR < 0.2), the rats were subjected to the frequency discrimination test, during which the acoustic stimuli were presented in the same manner as in the acclimation and the suppression training and each stimulus was tested for 5–6 sessions, with one session per day. Foot shock was delivered only if the SR for the speaker off period was >0.2. During the drug treatment, we allowed nine sessions (three sessions each frequency) for the animals to establish new associations with changes in their tinnitus status if there were any and data collected from the first nine sessions of drug treatment were discarded. During the first few days, more foot shocks were triggered by the animals, which suggested the re-establishment of conditioned suppression. Furthermore, animals were tested every day for a further 18 sessions (six sessions for each stimulus) during the drug treatment period. This ensured that the animals had enough time to be reconditioned and to produce reliable responses.

If a rat did not have tinnitus, it would associate the silence period with the foot shock and the presentation of the stimuli had no meaning to it, therefore, its drinking activity would not be affected during the acoustic stimuli presentation periods. However, if a rat had tinnitus, it would hear its tinnitus during the silence period and associate its tinnitus, instead of the silence, with the foot shock. Therefore, a stimulus with sensory features resembling tinnitus during the testing session should act as a conditioned stimulus and produce greater suppression during the stimulus presentation period. Using this method, we have successfully induced and assessed tinnitus in rats in our laboratory and confirmed that the duration of tinnitus can last as long as 10 months after the acoustic trauma exposure, although the hearing loss is temporary (18–21).

### Criteria to identify tinnitus animals

Following the first tinnitus test and before the drug treatment, the frequency discrimination curve from each of the acoustic trauma-exposed animals was constructed for BBN, 20 and 32 kHz, respectively, and compared with the mean frequency discrimination curve from the sham group. The exposed animals with lower SRs that were clearly separated from the sham animals at two or more intensity levels measured were considered to have tinnitus. This procedure inevitably meant that the sample sizes for the different groups were unequal, which is potentially a problem for the statistical analysis of repeated measures data, e.g., using analysis of variance (ANOVA). For this reason, we did not employ repeated measures ANOVAs but rather, a linear mixed model (LMM) analysis with a restricted maximum likelihood procedure, because it does not assume a balanced design and also addresses the correlation structure of the repeated measures data (see below) (23–26).

### Statistical analysis

All data were tested for normality and homogeneity of variance, and a LMM analysis was undertaken using SPSS 22. Where these assumptions were violated, the data were square root transformed and re-tested. The SR data for tinnitus assessment were analyzed with a LMM analysis using a restricted maximum likelihood procedure (18–20, 23–25). LMM analyses were used in preference to repeated measures ANOVAs because of the problems caused by extensive autocorrelation in repeated measures data; LMM analyses model the covariance structure of the repeated measures data in order to address this problem (23–25). The data were analyzed with group (sham-vehicle, sham-drug, exposed-no tinnitus-drug, or exposed-tinnitus-drug) as a fixed factor and intensity as a repeated measure. Akaike’s Information Criterion (AIC) was used to determine the most appropriate covariance structure. Where appropriate, Bonferroni’s corrected *post hoc* tests were used to make pairwise comparisons. Results were considered significant if *P* ≤ 0.05. The number of animals with or without behavioral evidence of tinnitus was compared before, during, and after the drug administration using Chi-square tests.

## Results

**Figure 1:**
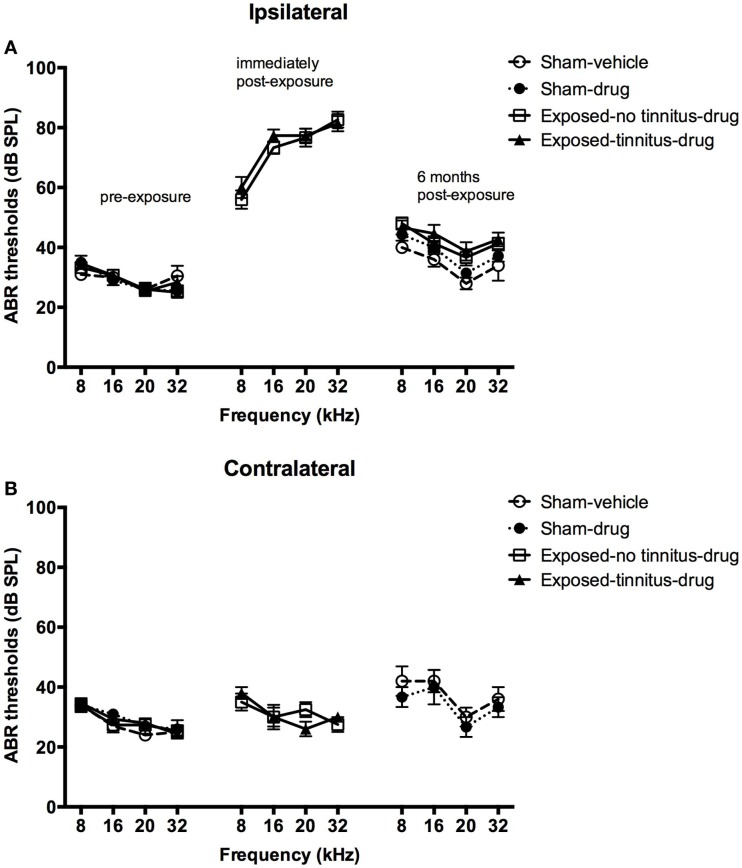
**ABR thresholds for the ipsilateral (A) and contralateral (B) ears of sham-vehicle, sham-acoustic, exposed-no tinnitus-drug, and exposed-tinnitus-drug animals pre-exposure, immediately post-exposure, and 6 months post-exposure, as a function of stimulus intensity in dB SPL and frequency in kHz**. Data are presented as means ± 1 SE.

In general, acoustic trauma resulted in a frequency-dependent increase in the ABR thresholds in the ipsilateral ear, which was similar for the tinnitus and no-tinnitus groups and which recovered partially over 6 months post-exposure. Acoustic trauma caused a significant increase in the ABR thresholds in the exposed animals as indicated by a significant group effect (*F*_3, 202.630_ = 2.874, *P* = 0.037) (Figures 1A,B). *Post hoc* tests revealed that there was no difference in the degree of ABR threshold elevation between the exposed-no tinnitus animals and the exposed-tinnitus animals (*P* = 1.000). The increase in the ABR thresholds was specifically in the ear ipsilateral to the acoustic trauma exposure and across all the frequencies tested, since there was a significant side effect (*F*_1, 240.459_ = 189.928, *P* = 0.0001) and a significant frequency effect (*F*_3, 517.500_ = 9.861, *P* = 0.0001). Moreover, the increase was also frequency-dependent as there were significant differences between all of the frequencies tested with larger increases at higher frequencies (8 vs 16 kHz, *P* = 0.29; 8 vs 20 kHz, *P* = 0.0001; 8 vs 32 kHz, *P* = 0.0001; 16 vs 20 kHz, *P* = 0.0001; 16 vs 32 kHz, *P* = 0.030; and 20 vs 32 kHz, *P* = 0.036). However, a significant side × frequency interaction indicated that the frequency-dependent increase in ABR threshold was specifically due to the ipsilateral ear (Figures 1A,B, middle panel). An overall significant time effect (*F*_2, 268.563_ = 245.389, *P* = 0.0001) and a side × time interaction (*F*_2, 253.408_ = 187.320, *P* = 0.0001) also confirmed an ipsilateral increase in ABR thresholds following acoustic trauma. Although there was a considerable recovery of the ABR thresholds at 6 months following acoustic trauma (Figures 1A,B, right panel), pairwise comparisons revealed a significant difference between the ABR thresholds before and immediately after acoustic trauma (*P* = 0.0001), immediately and at 6 months after acoustic trauma (*P* = 0.0001) as well as before and at 6 months after acoustic trauma (*P* = 0.0001).

**Figure 2:**
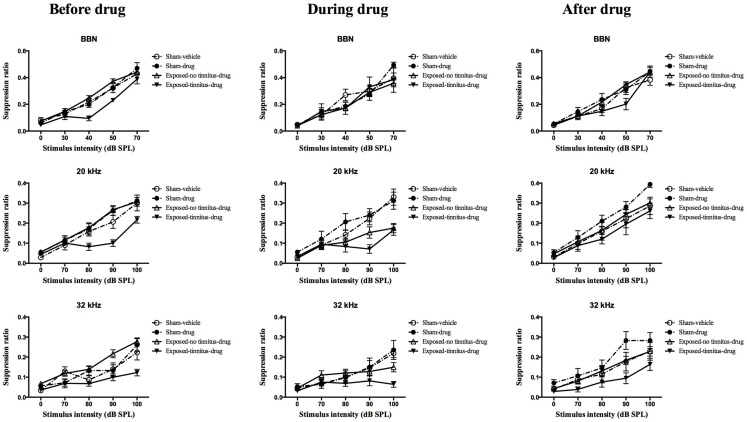
**Frequency discrimination curves for sham-vehicle (*n* = 10), sham-acoustic trauma (*n* = 10), exposed-no tinnitus-drug (*n* = 24, 22 and 20), and exposed-tinnitus-drug animals (*n* = 6, 8, and 10) in response to acoustic stimuli for BBN, 20 and 32 kHz tones before, during and after the drug administration**. Data are presented as means ± 1 SE.

At 1 month following the acoustic trauma, the animals underwent behavioral testing for the presence of tinnitus. After the completion of the test, the sham animals were randomly divided into two groups, vehicle and drug groups, and two mean frequency discrimination curves were constructed. A frequency discrimination curve was constructed for each of the exposed animals and compared with the two sham mean discrimination curves. The frequency discrimination curve showed a general increase in SR value with the increase in testing stimulus intensity, which reflects the increase in the discriminative nature between the testing stimulus and the conditioned stimulus (e.g., silence), i.e., the louder the testing stimulus is, the easier it is able to be distinguished from silence, therefore, the less suppression and the higher the SR value. Exposed animals were selected to become part of the exposed-tinnitus-drug group if two or more points on their frequency discrimination curve were clearly below the mean sham discrimination curves. The rest of the exposed animals were grouped as an exposed-no tinnitus-drug group. Based on this criterion, 6 animals were considered to experience tinnitus for BBN stimuli, 8 for 20 kHz stimuli, and 10 for 32 kHz stimuli. Among these animals, some of them had tinnitus at multiple frequencies while others had it only at a single frequency. Therefore, there were a total of 14 rats considered to have tinnitus. When the mean frequency discrimination curves were compared between these four groups, there was a significant group effect for BBN (*F*_3, 46.485_ = 5.155, *P* = 0.004), 20 kHz (*F*_2, 46.550_ = 4.386, *P* = 0.008) and 32 kHz (*F*_2, 46.592_ = 9.660, *P* = 0.000) stimuli (Figure 2, left panel). *Post hoc* tests revealed a significant difference between the exposed-tinnitus-drug group and exposed-no tinnitus-drug group for all three stimuli tested (BBN, *P* = 0.02; 20 kHz, *P* = 0.01; 32 kHz, *P* = 0.0001), between the exposed-tinnitus-drug group and sham-drug group for 20 kHz stimuli (*P* = 0.034) and between the exposed-tinnitus-drug group and sham-vehicle group for 32 kHz stimuli (*P* = 0.039).

In order to test whether the combination of THC and CBD could affect the perception of tinnitus in rats, the drugs were injected every day, 30 min before the tinnitus behavioral testing. During the administration of THC and CBD, there was a noticeable number of animals from the exposed-no tinnitus-drug group exhibiting greater lick suppression behavior in reaction to the presentation of the stimuli (Figure 2, middle panel). When the mean frequency discrimination curves from the four groups were compared, there was a significant group effect for 20 kHz stimuli (*F*_3, 45.006_ = 6.346, *P* = 0.001) and a significant group × intensity interaction for 32 kHz (*F*_12, 71.752_ = 1.902, *P* = 0.048), but there was no group effect for BBN. *Post hoc* tests revealed that when presented with 20 kHz tones, the mean frequency discrimination curve for the exposed-no tinnitus-drug group was significantly shifted downward and there was a significant difference between the exposed-no tinnitus-drug and sham-drug groups (*P* = 0.009). Moreover, the difference between the exposed-no tinnitus-drug and exposed-tinnitus-drug groups had disappeared (*P* = 1.000), which suggests that some animals from the exposed-no tinnitus-drug group had developed tinnitus while receiving THC and CBD. Although there was no significant group effect when 32 kHz stimuli were presented, the significant group × intensity interaction indicated that animals from different groups reacted differently to different intensities of the 32 kHz tones. A close inspection of the frequency discrimination curves (Figure 2, middle panel, third row) revealed that both the exposed-no tinnitus-drug and exposed-tinnitus-drug groups produced more lick suppression when the 32 kHz tone was presented at 100 dB SPL.

To find out whether THC and CBD would have any long-lasting effects on the animals’ tinnitus-like behavior, the animals were given a 2-week washout period during which the drug administration was stopped and the animals had free access to water and food. The tinnitus testing resumed after the washout period and there was only a significant group effect for 32 kHz tones (*F*_3, 48.581_ = 3.870, *P* = 0.015), but not BBN or 20 kHz tones. This significant group effect was due to the difference between the exposed-tinnitus-drug and sham-drug groups (*P* = 0.011).

**Figure 3:**
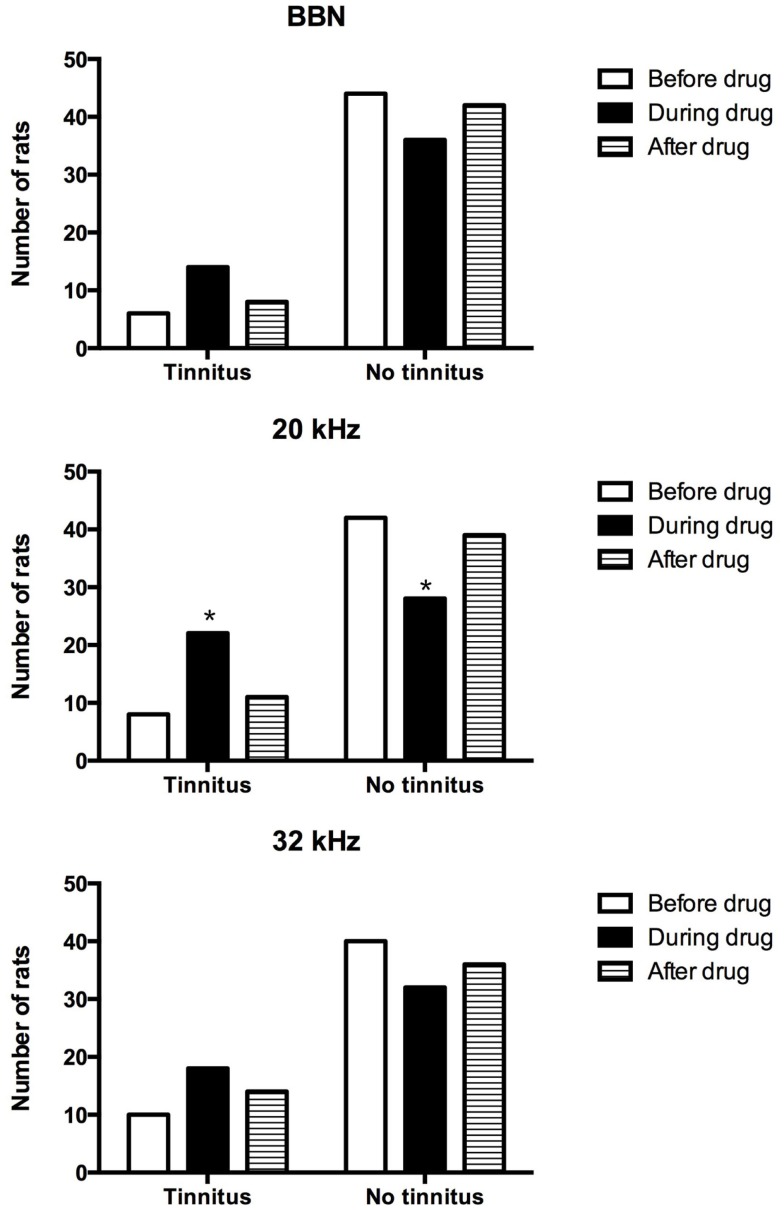
**Number of tinnitus and no tinnitus animals following acoustic trauma before, during, and after the drug administration**.

In addition, the proportion of acoustic trauma-exposed animals that had behavioral signs of tinnitus was compared before, during, and after the drug administration for BBN, 20 or 32 kHz stimuli presentations (Figure 3). Although more animals displayed behavioral evidence of tinnitus during the administration of THC and CBD for all three stimuli tested, a significant increase in the number of tinnitus animals was evident only for 20 kHz stimuli (χ^2^ = 10.94, df = 2, *P* = 0.004). There was no difference in the number of tinnitus animals before and after the drug administration.

## Discussion

Our results showed that following acoustic trauma, only a proportion of animals developed tinnitus and the rest did not. However, the combination of THC and CBD reversibly increased the number of tinnitus animals in the exposed, but not the sham groups, which suggests that THC and CBD may promote the perception of tinnitus if there is pre-existing hearing damage.

It has been shown that not every animal exposed to acoustic trauma develops tinnitus, with the reported tinnitus-induction rate varying from 30 to 80% [see Ref. (27) for a review]. Whether an animal develops tinnitus or not seems to be not directly correlated with the degree of hearing loss either immediately after the acoustic trauma exposure or a few months later [see Ref. (27) for a review]. In the present study, all of our rats exhibited elevated ABR thresholds across a range of frequencies immediately after the acoustic trauma. It has been reported that exposure to loud tones at 10 kHz resulted in an immediate hearing loss across a wide range of frequencies both below and higher (i.e., 6–24 kHz) than the exposed frequency using compound action potential audiograms (28), which is in agreement with our observations. However, it is believed that the maximum hearing loss following exposure to loud tones usually occurred at half an octave above the exposed frequency (29). However, in the present study the maximum hearing loss was at 32 kHz, which is a full octave above the exposed frequency of 16 kHz. This might be due to the presence of harmonic distortion of the 16 kHz tone. However, this harmonic tone at 32 kHz was measured 30 dB below the 16 kHz tones, which should be less likely to cause greater hearing loss, although unexpected damage could still occur due to the increased susceptibility of hair cells in the higher frequency regions to free radicals (30). It might also be necessary to measure hearing loss at 24 kHz following exposure to 16 kHz tones in order to confirm whether a greater hearing loss would occur at half an octave above the exposed frequency. Nevertheless, the hearing loss recovered substantially at 6 months following exposure. Although the ABR thresholds in the exposed animals did not completely return to the pre-exposure level, they were not different from the sham animals tested in parallel. Therefore, the slightly elevated ABR thresholds at the end of the experiment might have been due to age-related changes, although it seems less likely to be the case given that the age of our rats (9–10 months old) was a few months younger than the age of the Wistar rats (12–14 months old) showing age-related hearing loss (31). Nevertheless, the fact that our exposed animals had similar degrees of acute hearing loss and recovery and only some of them developed tinnitus, suggests that tinnitus development might not be reflected by the gross changes in ABR thresholds. Schaette and McAlpine (32) reported that human tinnitus subjects could have a normal audiogram and a normal amplitude of the centrally generated ABR wave V, but a significantly reduced amplitude of the auditory nerve-generated ABR wave I, which suggests a “hidden hearing loss” in these tinnitus patients. In addition, the relationship between altered ABR waveforms and tinnitus has also been studied in animals models (33, 34), with an increase in early ABR wave amplitude up to N3 (33) and both increases (33) and decreases (34) in latencies reported. In this study, the ABR was not measured either at 1 month post-exposure or during the drug treatment, when changes in the animal’s tinnitus status occurred. Therefore, it is impossible to make valid correlations using the currently available ABR results. Future studies are needed to further analyze changes in the different components of the ABR waves in animals with tinnitus.

Among the animals that exhibited behavioral evidence of tinnitus, i.e., the downward shift of the frequency discrimination curve, tinnitus manifested at different frequencies, with some animals experiencing it at multiple frequencies and within the same animal, there were fluctuations of tinnitus-like behavior in response to specific frequencies at different time points following the acoustic trauma. These observations are generally in agreement with our previous publications and those of others (35–38). Following the administration of THC and CBD, exposed-no tinnitus-animals showed increased suppression during the drug treatment for the 20 kHz stimulus and to a lesser extent, for the 32 kHz stimulus and a separate analysis looking at the proportion of animals experiencing tinnitus showed that there were more tinnitus animals during the drug treatment in response to the 20 kHz stimulus. Taken together, the results indicated that more animals shift from no-tinnitus to tinnitus status in the exposed-no tinnitus group, which suggests that THC and CBD may promote or enhance the perception of tinnitus in animals. However, following a 2-week washout period, this effect disappeared for 20 kHz stimuli. In addition, animals in the sham-drug group showed an up-shift in the suppression curve for both 20 and 32 kHz stimuli. The explanation for these effects is unknown; however, because delta-9-THC has a long half-life and is sequestered in fat (39), it is possible that this is due to some kind of delayed therapeutic effect. Perhaps another washout test at a later time point could provide more conclusive results.

Due to the fact that the number of animals exhibiting behavioral signs of tinnitus was similar before and after drug treatment, but significantly increased during drug treatment, this increase in the number of tinnitus animals cannot be explained simply by tinnitus fluctuation. In addition, we also observed greater lick suppression in response to the BBN, which has been suggested to be due to hearing loss (37). In this study, this increase in suppression in response to the BBN was only evident at 1 month post-exposure, but not at later time points, which might reflect temporary hearing loss following acoustic trauma. However, the ABR was not measured at 1 month post-exposure in this study; therefore, a definitive conclusion could not be drawn.

It has been shown that acoustic trauma results in neuronal hyperactivity in different areas of the brain including the cochlear nucleus, the inferior colliculus, the medial geniculate body, and the auditory cortex (5, 40–44). The hyperactivity, at least in the DCN, has been attributed to a decrease of GABAergic inhibition (45) and the burst firing of the fusiform cells (46). One type of GABAergic interneuron in the DCN is the cartwheel cells, which strongly inhibit fusiform cells through feed-forward inhibition (47). Presynaptic CB_1_ receptors have been found at the terminals of parallel fibers synapsing with the cartwheel cells and activating the presynaptic CB_1_ receptors, as a result of either the sustained firing of cartwheel cells or the application of a CB_1_ receptor agonist, significantly reducing the synaptic strength (15, 48). Therefore, it is conceivable that the activation of CB_1_ receptors on presynaptic terminals resulted in a decrease in GABA release from the cartwheel cells, which in turn resulted in a reduction in inhibition of the fusiform cells. It is interesting that this effect was only observed in animals that had been exposed to acoustic trauma, but not in sham animals. It has been reported that somatosensory input transmitted by parallel fibers produced a suppression-dominant effect on auditory processes in normal animals, but this effect was shifted to enhancement in exposed-tinnitus animals and to a much less extent in exposed-no tinnitus animals (49). Although this shift to enhancement is less pronounced in exposed no tinnitus animals, the cannabinoids might be able to increase this enhancement effect to the level comparable to that in exposed tinnitus animals. However, cannabinoids might not be able to shift the suppression-dominant effect to enhancement in sham animals. Having said this, it must be appreciated that the drug administration in this study was systemic, and therefore, the actions of THC and CBD cannot be attributed solely to the cochlear nucleus or even the central auditory system; in fact, the effects of these cannabinoids on any area of the CNS, including the limbic system that projects to the central auditory system, could conceivably have contributed to the observed effects on tinnitus-related behavior. The other issue that must be noted is that although delta-9-THC is a partial agonist at CB_1_ receptors, CBD can act as a partial CB_1_ antagonist (50), and it is impossible to know the net effect of these two drugs, even in the cochlear nucleus. Furthermore, previous studies have demonstrated that acute and chronic dose regimens with cannabinoid drugs can have quite different effects. For example, Okine et al. (51) reported that chronic pre-treatment with URB597, and inhibitor of fatty acid amide hydrolase, a key enzyme in the metabolism of the endocannabinoid, anandamide, had no effect on inflammatory pain behavior in rats, whereas a single dose significantly reduced it. It is therefore quite conceivable that we might have observed different results with acute dosing of delta-9-THC/CBD.

In addition, endocannabinoids in the central nucleus of the amygdala have been implicated in short-term adaptation of the conditioned fear response, and the CB_1_ receptor antagonist AM251 increased the fear response (52). Because systemic injections were used to deliver the drugs in this study, it could be argued that changes in the frequency discrimination curve might not reflect the perception of tinnitus but rather changes in the fear response through the drugs’ effects on the amygdala. However, if this was the case, cannabinoids would facilitate the adaptation of the conditioned fear response and result in an upward shift of the curve rather than the downward shift observed in the exposed no tinnitus animals. A close inspection of the curves did reveal a slight upward shift of the curve in the sham-drug animals in response to 20 kHz tones during the drug administration and in response to 20 and 32 kHz tones after drug administration, which suggests that adaptation of the conditioned fear response might have occurred in our animals. However, this adaptation was not enough to affect the greater lick suppression in animals with tinnitus.

Although *Cannabis* is used by some tinnitus sufferers to relieve their condition, our results, consistent with our previous study using the salicylate model (17), suggest that cannabinoids, such as delta-9-THC and CBD, may actually aggravate tinnitus (53). This might be predicted from the work of Zhao et al. (16), which suggested that the net effect of activation of CB_1_ receptors in the DCN might be to increase the excitation of fusiform cells, thus exacerbating neuronal hyperactivity.

## Conflict of Interest Statement

The authors declare that the research was conducted in the absence of any commercial or financial relationships that could be construed as a potential conflict of interest.
